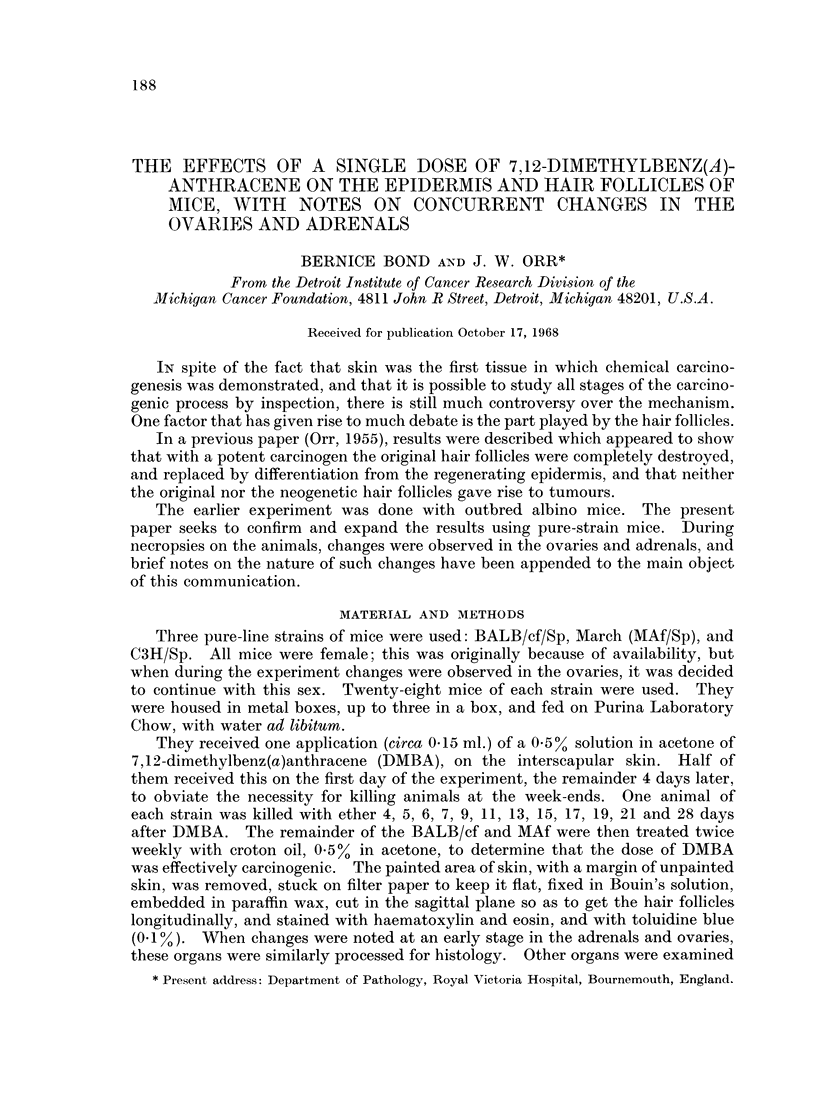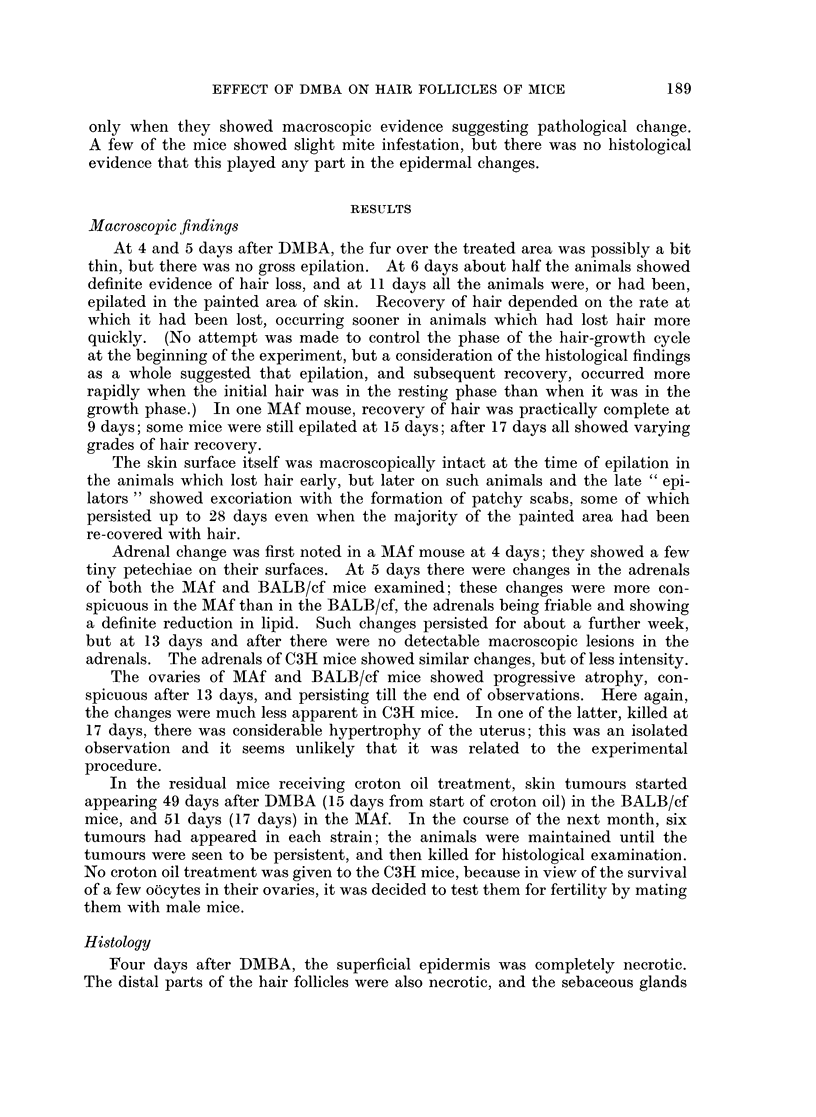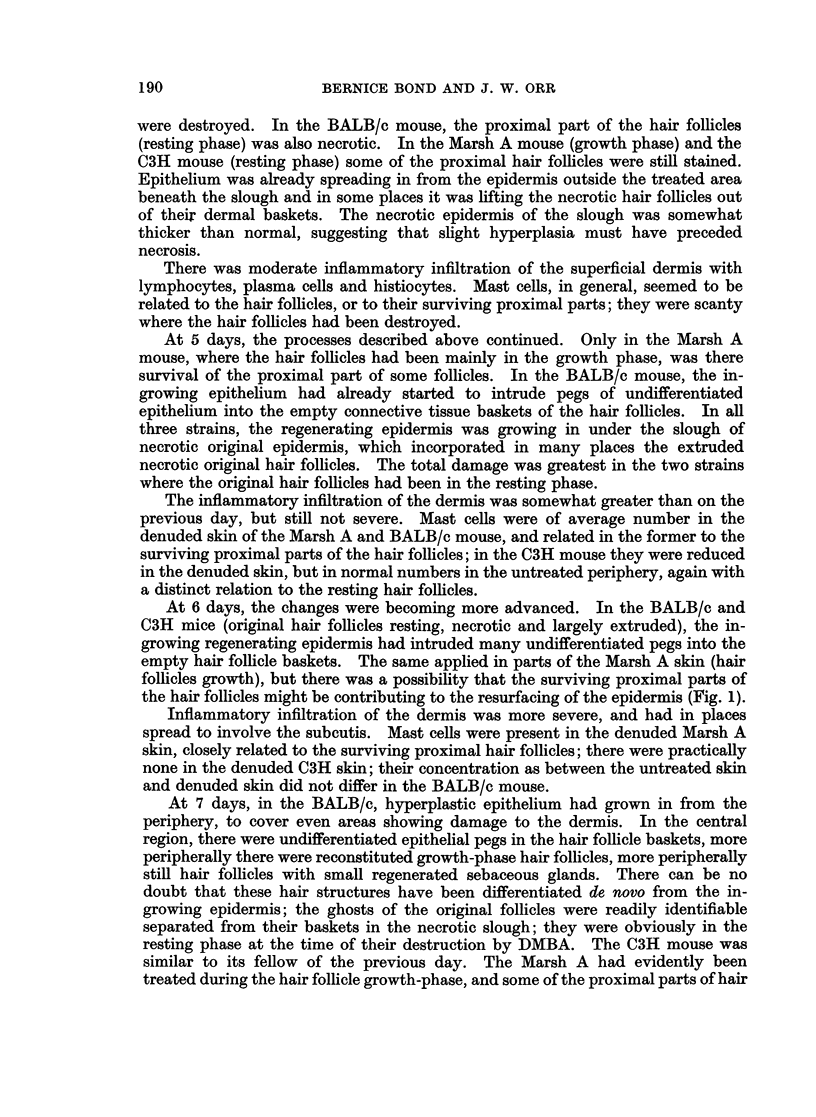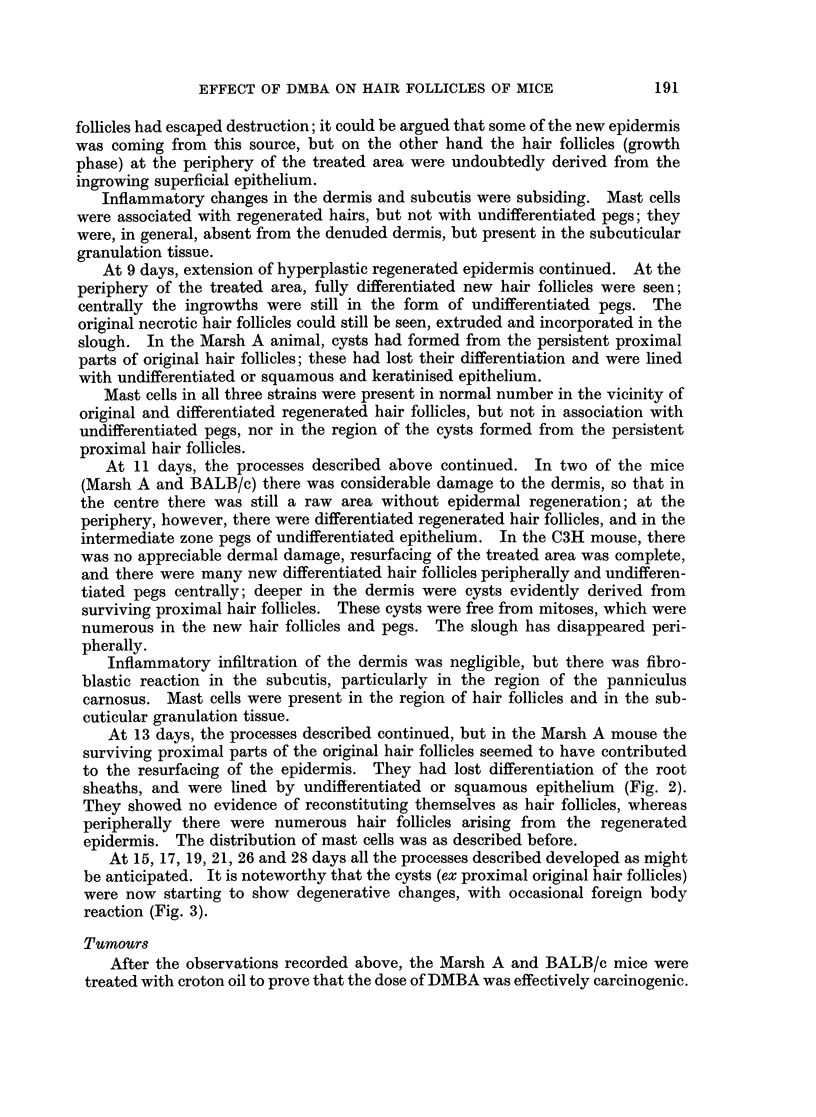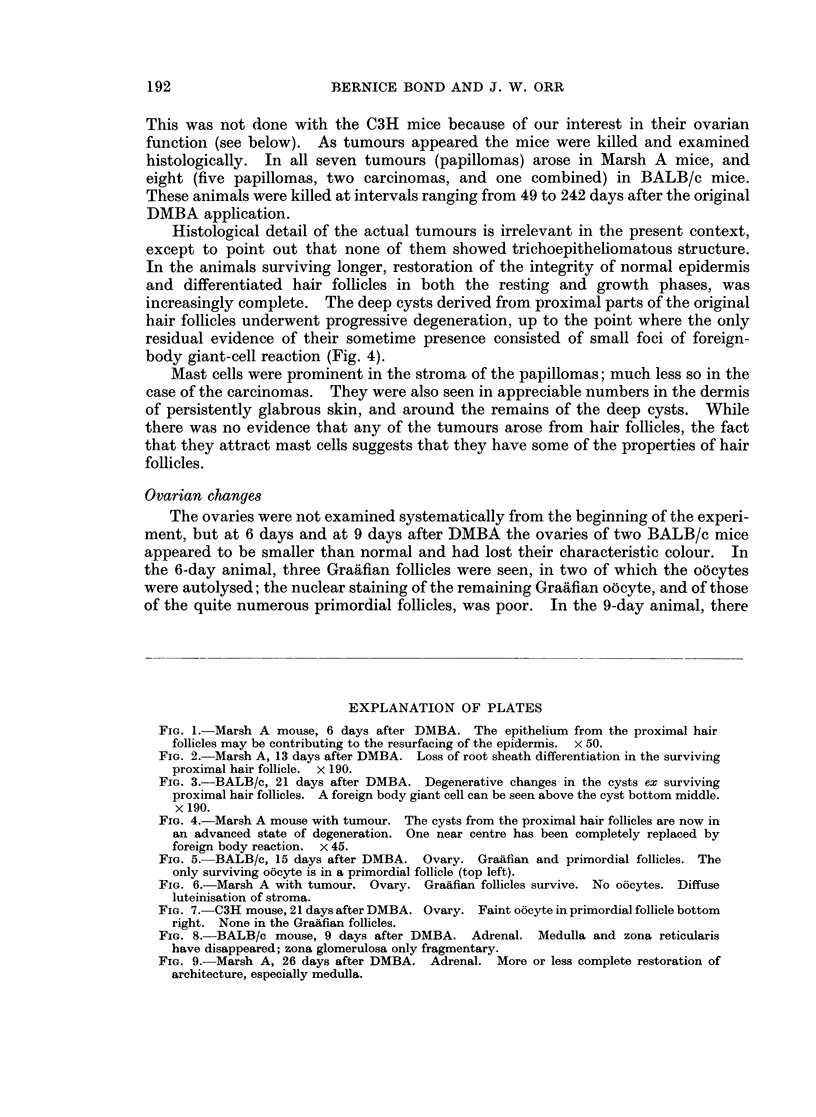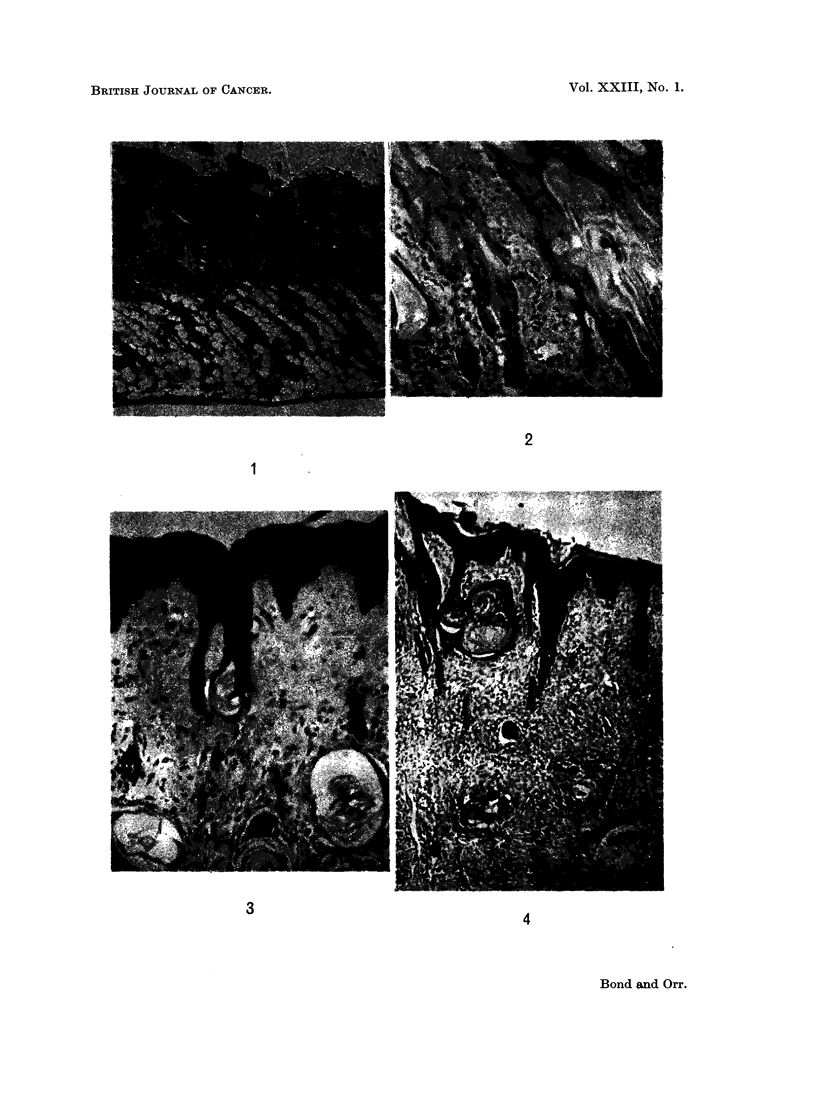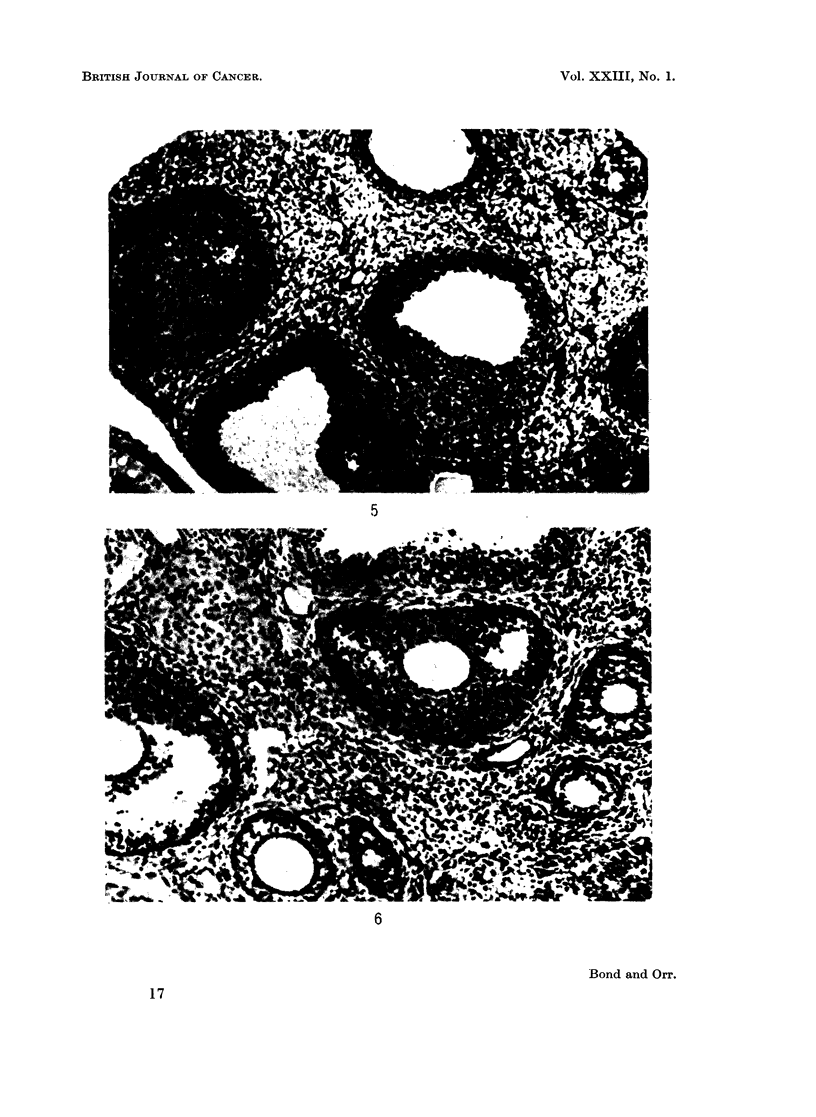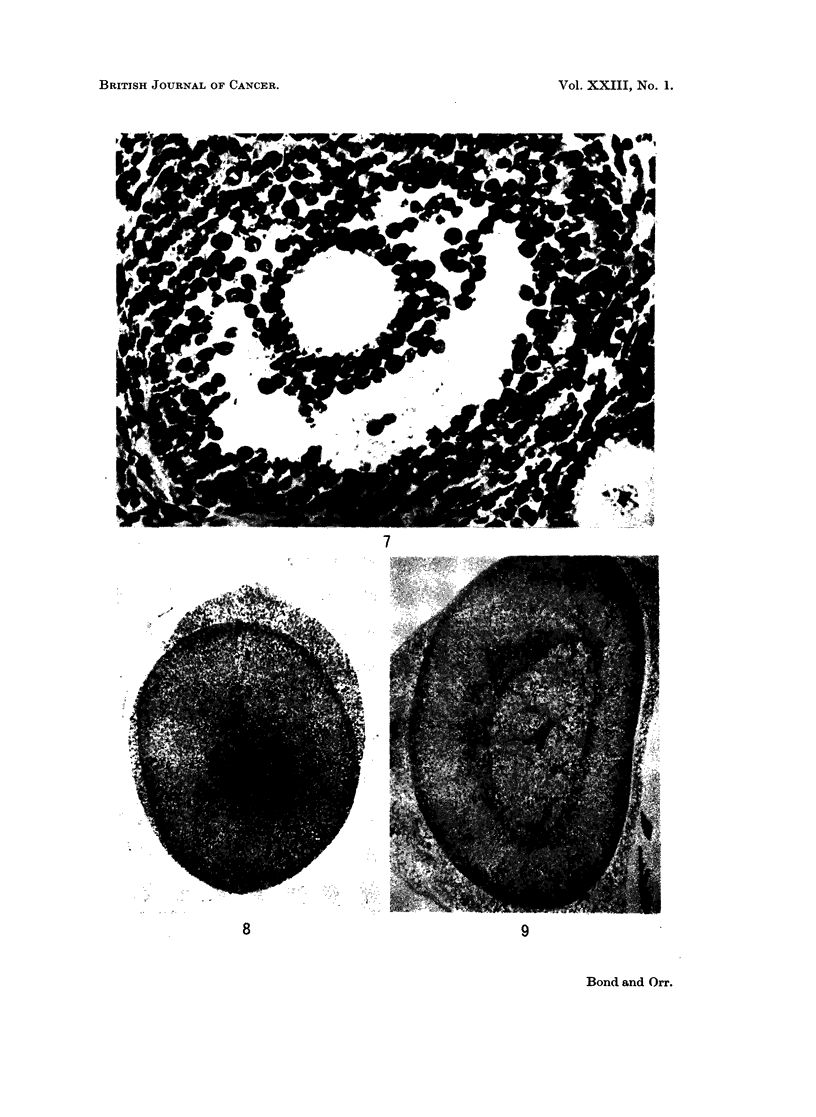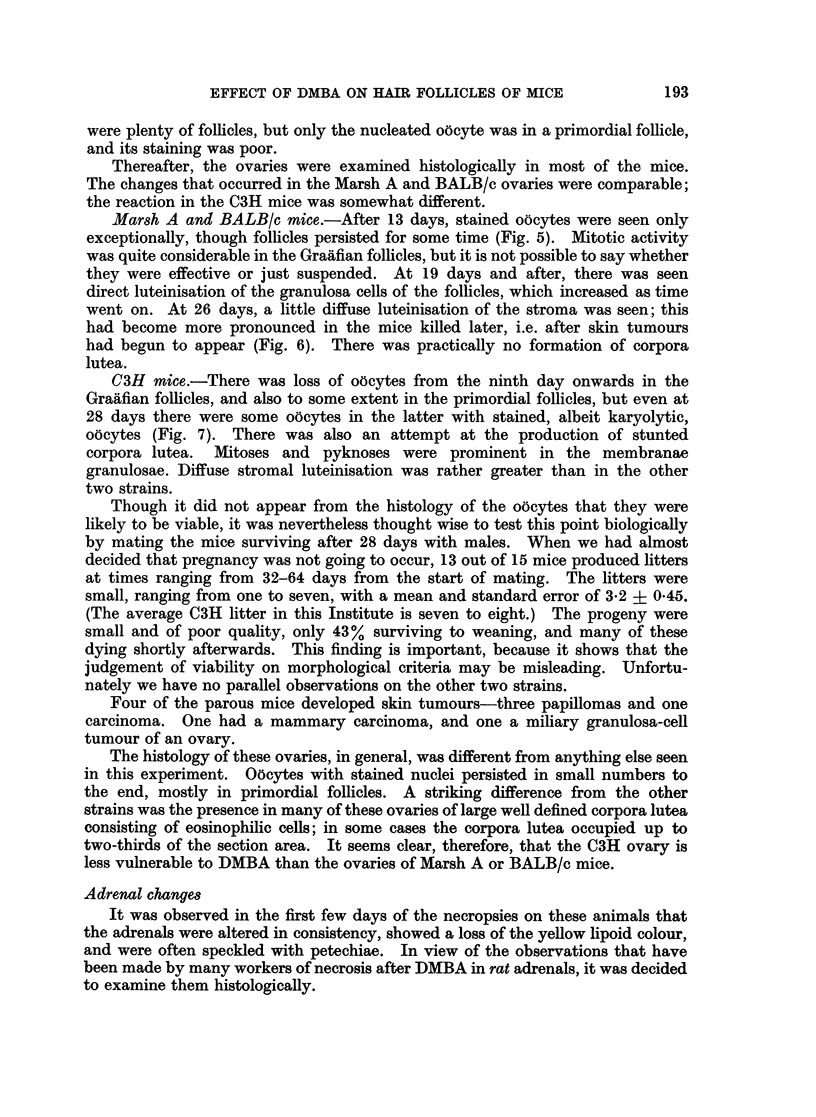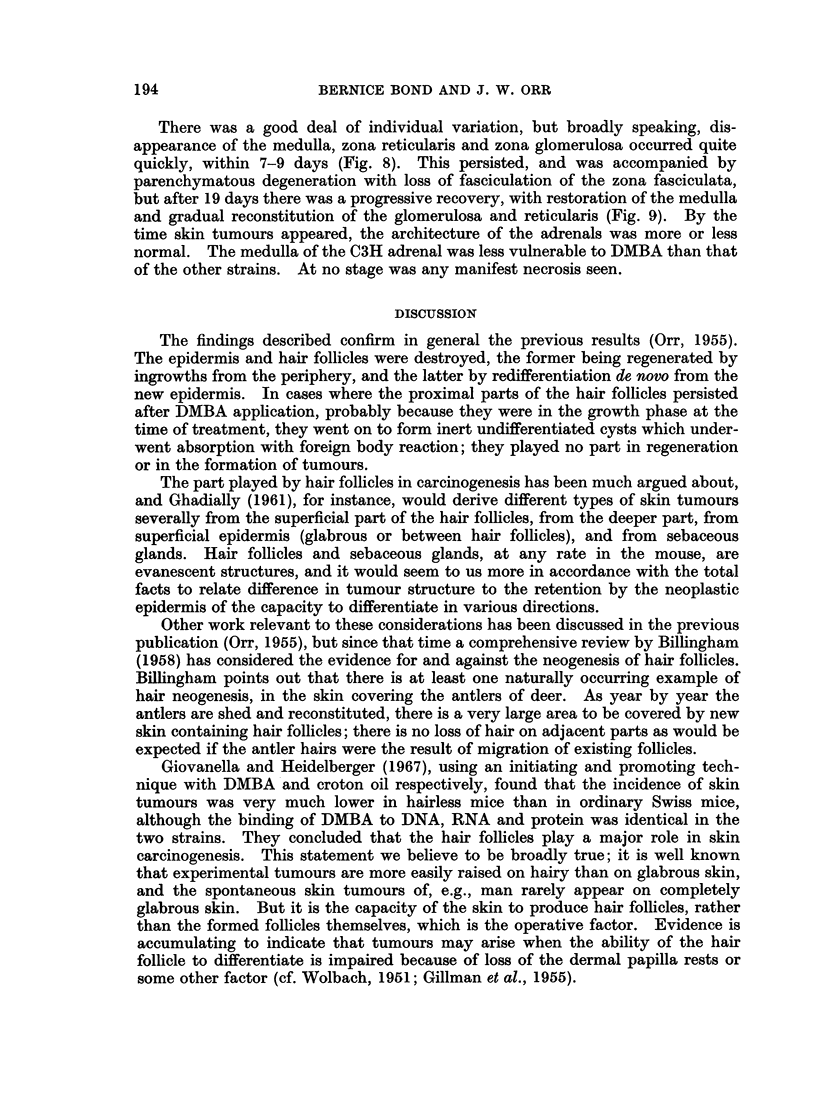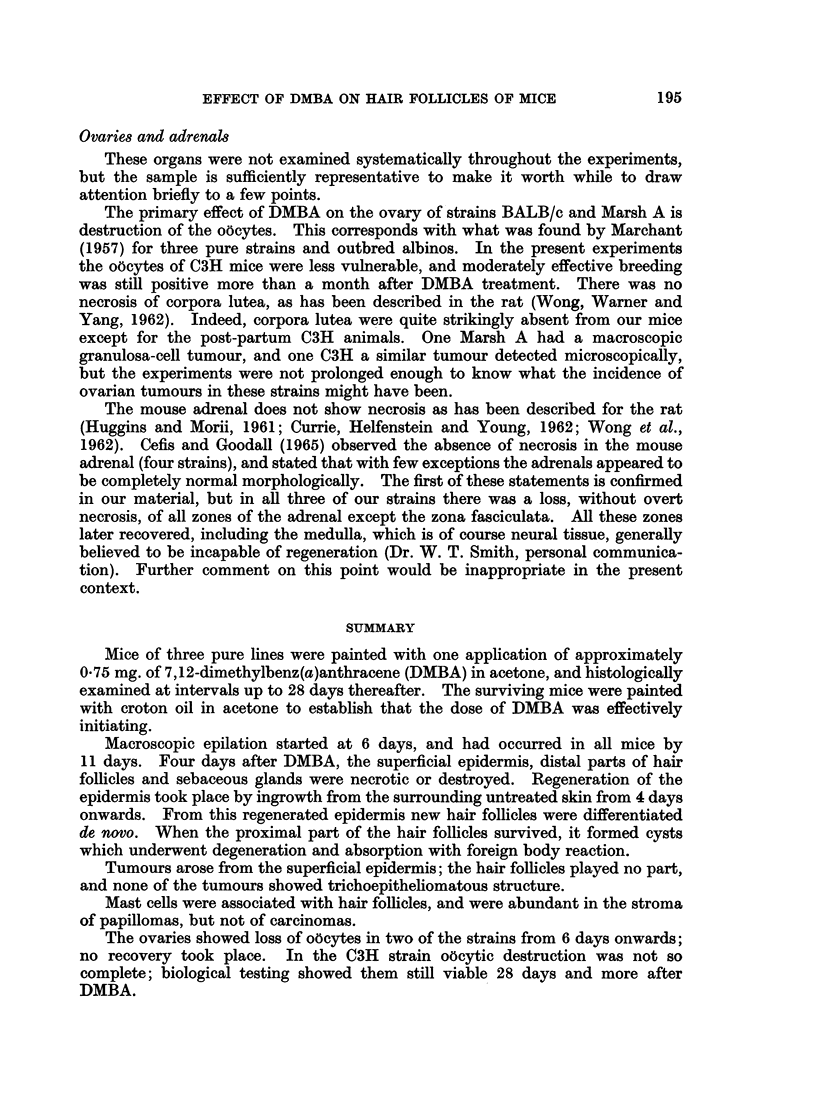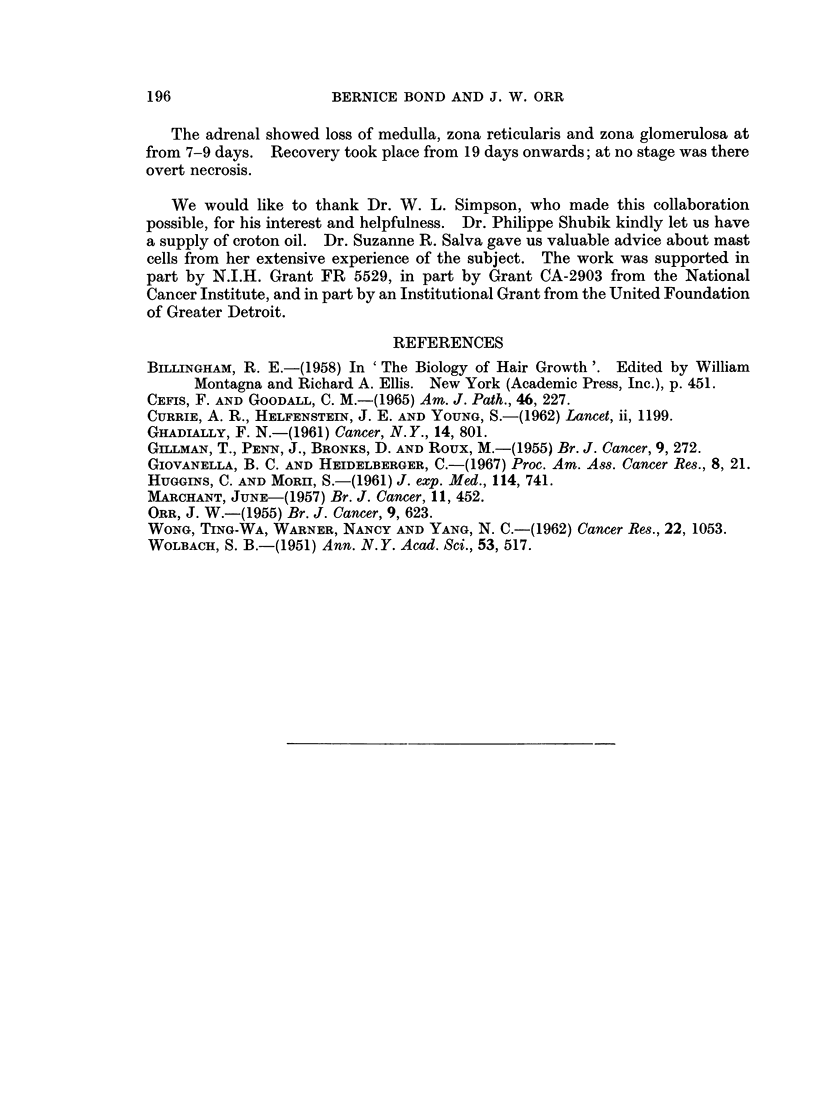# The effects of a single dose of 7,12-dimethylbenz(a)-anthracene on the epidermis and hair follicles of mice, with notes on concurrent changes in the ovaries and adrenals.

**DOI:** 10.1038/bjc.1969.27

**Published:** 1969-03

**Authors:** B. Bond, J. W. Orr

## Abstract

**Images:**


### THE EFFECTS OF A SINGLE DOSE OF 7,12-DIMETHYLBENZ(*A*)-ANTHRACENE ON THE EPIDERMIS AND HAIR FOLLICLES OF MICE, WITH NOTES ON CONCURRENT CHANGES IN THE OVARIES AND ADRENALS

BERNICE BOND AND J. W. ORR*

*From the Detroit Institute of Cancer Research Division of the Michigan Cancer Foundation,* 4811 *John R Street, Detroit, Michigan* 48201, *U.S.A.*

Received for publication October 17, 1968

IN spite of the fact that skin was the first tissue in which chemical carcinogenesis was demonstrated, and that it is possible to study all stages of the carcinogenic process by inspection, there is still much controversy over the mechanism. One factor that has given rise to much debate is the part played by the hair follicles.

In a previous paper (Orr, 1955), results were described which appeared to show that with a potent carcinogen the original hair follicles were completely destroyed, and replaced by differentiation from the regenerating epidermis, and that neither the original nor the neogenetic hair follicles gave rise to tumours.

The earlier experiment was done with outbred albino mice. The present paper seeks to confirm and expand the results using pure-strain mice. During necropsies on the animals, changes were observed in the ovaries and adrenals, and brief notes on the nature of such changes have been appended to the main object of this communication.

### MATERIAL AND METHODS

Three pure-line strains of mice were used: BALB/cf/Sp, March (MAf/Sp), and C3H/Sp. All mice were female; this was originally because of availability, but when during the experiment changes were observed in the ovaries, it was decided to continue with this sex. Twenty-eight mice of each strain were used. They were housed in metal boxes, up to three in a box, and fed on Purina Laboratory Chow, with water *ad libitum.*

They received one application (*circa* 0·15 ml.) of a 0·5% solution in acetone of 7,12-dimethylbenz(*a*)anthracene (DMBA), on the interscapular skin. Half of them received this on the first day of the experiment, the remainder 4 days later, to obviate the necessity for killing animals at the week-ends. One animal of each strain was killed with ether 4, 5, 6, 7, 9, 11, 13, 15, 17, 19, 21 and 28 days after DMBA. The remainder of the BALB/cf and MAf were then treated twice weekly with croton oil, 0·5% in acetone, to determine that the dose of DMBA was effectively carcinogenic. The painted area of skin, with a margin of unpainted skin, was removed, stuck on filter paper to keep it flat, fixed in Bouin's solution, embedded in paraffin wax, cut in the sagittal plane so as to get the hair follicles longitudinally, and stained with haematoxylin and eosin, and with toluidine blue (0·1%). When changes were noted at an early stage in the adrenals and ovaries, these organs were similarly processed for histology. Other organs were examined

* Present address: Department of Pathology, Royal Victoria Hospital, Bournemouth, England.

only when they showed macroscopic evidence suggesting pathological change. A few of the mice showed slight mite infestation, but there was no histological evidence that this played any part in the epidermal changes.

### RESULTS

### *Macroscopic findings*

At 4 and 5 days after DMBA, the fur over the treated area was possibly a bit thin, but there was no gross epilation. At 6 days about half the animals showed definite evidence of hair loss, and at 11 days all the animals were, or had been, epilated in the painted area of skin. Recovery of hair depended on the rate at which it had been lost, occurring sooner in animals which had lost hair more quickly. (No attempt was made to control the phase of the hair-growth cycle at the beginning of the experiment, but a consideration of the histological findings as a whole suggested that epilation, and subsequent recovery, occurred more rapidly when the initial hair was in the resting phase than when it was in the growth phase.) In one MAf mouse, recovery of hair was practically complete at 9 days; some mice were still epilated at 15 days; after 17 days all showed varying grades of hair recovery.

The skin surface itself was macroscopically intact at the time of epilation in the animals which lost hair early, but later on such animals and the late " epilators " showed excoriation with the formation of patchy scabs, some of which persisted up to 28 days even when the majority of the painted area had been re-covered with hair.

Adrenal change was first noted in a MAf mouse at 4 days; they showed a few tiny petechiae on their surfaces. At 5 days there were changes in the adrenals of both the MAf and BALB/cf mice examined; these changes were more conspicuous in the MAf than in the BALB/cf, the adrenals being friable and showing a definite reduction in lipid. Such changes persisted for about a further week, but at 13 days and after there were no detectable macroscopic lesions in the adrenals. The adrenals of C3H mice showed similar changes, but of less intensity.

The ovaries of MAf and BALB/cf mice showed progressive atrophy, conspicuous after 13 days, and persisting till the end of observations. Here again, the changes were much less apparent in C3H mice. In one of the latter, killed at 17 days, there was considerable hypertrophy of the uterus; this was an isolated observation and it seems unlikely that it was related to the experimental procedure.

In the residual mice receiving croton oil treatment, skin tumours started appearing 49 days after DMBA (15 days from start of croton oil) in the BALB/cf mice, and 51 days (17 days) in the MAf. In the course of the next month, six tumours had appeared in each strain; the animals were maintained until the tumours were seen to be persistent, and then killed for histological examination. No croton oil treatment was given to the C3H mice, because in view of the survival of a few oöcytes in their ovaries, it was decided to test them for fertility by mating them with male mice.

### *Histology*

Four days after DMBA, the superficial epidermis was completely necrotic. The distal parts of the hair follicles were also necrotic, and the sebaceous glands were destroyed. In the BALB/c mouse, the proximal part of the hair follicles (resting phase) was also necrotic. In the Marsh A mouse (growth phase) and the C3H mouse (resting phase) some of the proximal hair follicles were still stained. Epithelium was already spreading in from the epidermis outside the treated area beneath the slough and in some places it was lifting the necrotic hair follicles out of their dermal baskets. The necrotic epidermis of the slough was somewhat thicker than normal, suggesting that slight hyperplasia must have preceded necrosis.

There was moderate inflammatory infiltration of the superficial dermis with lymphocytes, plasma cells and histiocytes. Mast cells, in general, seemed to be related to the hair follicles, or to their surviving proximal parts; they were scanty where the hair follicles had been destroyed.

At 5 days, the processes described above continued. Only in the Marsh A mouse, where the hair follicles had been mainly in the growth phase, was there survival of the proximal part of some follicles. In the BALB/c mouse, the ingrowing epithelium had already started to intrude pegs of undifferentiated epithelium into the empty connective tissue baskets of the hair follicles. In all three strains, the regenerating epidermis was growing in under the slough of necrotic original epidermis, which incorporated in many places the extruded necrotic original hair follicles. The total damage was greatest in the two strains where the original hair follicles had been in the resting phase.

The inflammatory infiltration of the dermis was somewhat greater than on the previous day, but still not severe. Mast cells were of average number in the denuded skin of the Marsh A and BALB/c mouse, and related in the former to the surviving proximal parts of the hair follicles; in the C3H mouse they were reduced in the denuded skin, but in normal numbers in the untreated periphery, again with a distinct relation to the resting hair follicles.

At 6 days, the changes were becoming more advanced. In the BALB/c and C3H mice (original hair follicles resting, necrotic and largely extruded), the ingrowing regenerating epidermis had intruded many undifferentiated pegs into the empty hair follicle baskets. The same applied in parts of the Marsh A skin (hair follicles growth), but there was a possibility that the surviving proximal parts of the hair follicles might be contributing to the resurfacing of the epidermis (Fig. 1).

Inflammatory infiltration of the dermis was more severe, and had in places spread to involve the subcutis. Mast cells were present in the denuded Marsh A skin, closely related to the surviving proximal hair follicles; there were practically none in the denuded C3H skin; their concentration as between the untreated skin and denuded skin did not differ in the BALB/c mouse.

At 7 days, in the BALB/c, hyperplastic epithelium had grown in from the periphery, to cover even areas showing damage to the dermis. In the central region, there were undifferentiated epithelial pegs in the hair follicle baskets, more peripherally there were reconstituted growth-phase hair follicles, more peripherally still hair follicles with small regenerated sebaceous glands. There can be no doubt that these hair structures have been differentiated *de novo* from the ingrowing epidermis; the ghosts of the original follicles were readily identifiable separated from their baskets in the necrotic slough; they were obviously in the resting phase at the time of their destruction by DMBA. The C3H mouse was similar to its fellow of the previous day. The Marsh A had evidently been treated during the hair follicle growth-phase, and some of the proximal parts of hair follicles had escaped destruction; it could be argued that some of the new epidermis was coming from this source, but on the other hand the hair follicles (growth phase) at the periphery of the treated area were undoubtedly derived from the ingrowing superficial epithelium.

Inflammatory changes in the dermis and subcutis were subsiding. Mast cells were associated with regenerated hairs, but not with undifferentiated pegs; they were, in general, absent from the denuded dermis, but present in the subcuticular granulation tissue.

At 9 days, extension of hyperplastic regenerated epidermis continued. At the periphery of the treated area, fully differentiated new hair follicles were seen; centrally the ingrowths were still in the form of undifferentiated pegs. The original necrotic hair follicles could still be seen, extruded and incorporated in the slough. In the Marsh A animal, cysts had formed from the persistent proximal parts of original hair follicles; these had lost their differentiation and were lined with undifferentiated or squamous and keratinised epithelium.

Mast cells in all three strains were present in normal number in the vicinity of original and differentiated regenerated hair follicles, but not in association with undifferentiated pegs, nor in the region of the cysts formed from the persistent proximal hair follicles.

At 11 days, the processes described above continued. In two of the mice (Marsh A and BALB/c) there was considerable damage to the dermis, so that in the centre there was still a raw area without epidermal regeneration; at the periphery, however, there were differentiated regenerated hair follicles, and in the intermediate zone pegs of undifferentiated epithelium. In the C3H mouse, there was no appreciable dermal damage, resurfacing of the treated area was complete, and there were many new differentiated hair follicles peripherally and undifferentiated pegs centrally; deeper in the dermis were cysts evidently derived from surviving proximal hair follicles. These cysts were free from mitoses, which were numerous in the new hair follicles and pegs. The slough has disappeared peripherally.

Inflammatory infiltration of the dermis was negligible, but there was fibroblastic reaction in the subcutis, particularly in the region of the panniculus carnosus. Mast cells were present in the region of hair follicles and in the subcuticular granulation tissue.

At 13 days, the processes described continued, but in the Marsh A mouse the surviving proximal parts of the original hair follicles seemed to have contributed to the resurfacing of the epidermis. They had lost differentiation of the root sheaths, and were lined by undifferentiated or squamous epithelium (Fig. 2). They showed no evidence of reconstituting themselves as hair follicles, whereas peripherally there were numerous hair follicles arising from the regenerated epidermis. The distribution of mast cells was as described before.

At 15, 17, 19, 21, 26 and 28 days all the processes described developed as might be anticipated. It is noteworthy that the cysts (*ex* proximal original hair follicles) were now starting to show degenerative changes, with occasional foreign body reaction (Fig. 3).

*Tumours*

After the observations recorded above, the Marsh A and BALB/c mice were treated with croton oil to prove that the dose of DMBA was effectively carcinogenic.

This was not done with the C3H mice because of our interest in their ovarian function (see below). As tumours appeared the mice were killed and examined histologically. In all seven tumours (papillomas) arose in Marsh A mice, and eight (five papillomas, two carcinomas, and one combined) in BALB/c mice. These animals were killed at intervals ranging from 49 to 242 days after the original DMBA application.

Histological detail of the actual tumours is irrelevant in the present context, except to point out that none of them showed trichoepitheliomatous structure. In the animals surviving longer, restoration of the integrity of normal epidermis and differentiated hair follicles in both the resting and growth phases, was increasingly complete. The deep cysts derived from proximal parts of the original hair follicles underwent progressive degeneration, up to the point where the only residual evidence of their sometime presence consisted of small foci of foreign-body giant-cell reaction (Fig. 4).

Mast cells were prominent in the stroma of the papillomas; much less so in the case of the carcinomas. They were also seen in appreciable numbers in the dermis of persistently glabrous skin, and around the remains of the deep cysts. While there was no evidence that any of the tumours arose from hair follicles, the fact that they attract mast cells suggests that they have some of the properties of hair follicles.

*Ovarian changes*

The ovaries were not examined systematically from the beginning of the experiment, but at 6 days and at 9 days after DMBA the ovaries of two BALB/c mice appeared to be smaller than normal and had lost their characteristic colour. In the 6-day animal, three Graäfian follicles were seen, in two of which the oöcytes were autolysed; the nuclear staining of the remaining Graäfian oöcyte, and of those of the quite numerous primordial follicles, was poor. In the 9-day animal, there

---

EXPLANATION OF PLATES

Fig. 1.—Marsh A mouse, 6 days after DMBA. The epithelium from the proximal hair follicles may be contributing to the resurfacing of the epidermis. × 50.

Fig. 2.—Marsh A, 13 days after DMBA. Loss of root sheath differentiation in the surviving proximal hair follicle. × 190.

Fig. 3.—BALB/c, 21 days after DMBA. Degenerative changes in the cysts *ex* surviving proximal hair follicles. A foreign body giant cell can be seen above the cyst bottom middle. × 190.

Fig. 4.—Marsh A mouse with tumour. The cysts from the proximal hair follicles are now in an advanced state of degeneration. One near centre has been completely replaced by foreign body reaction. × 45.

Fig. 5.—BALB/c, 15 days after DMBA. Ovary. Graäfian and primordial follicles. The only surviving oöcyte is in a primordial follicle (top left).

Fig. 6.—Marsh A with tumour. Ovary. Graäfian follicles survive. No oöcytes. Diffuse luteinisation of stroma.

Fig. 7.—C3H mouse, 21 days after DMBA. Ovary. Faint oöcyte in primordial follicle bottom right. None in the Graäfian follicles.

Fig. 8.—BALB/c mouse, 9 days after DMBA. Adrenal. Medulla and zona reticularis have disappeared; zona glomerulosa only fragmentary.

Fig. 9.—Marsh A, 26 days after DMBA. Adrenal. More or less complete restoration of architecture, especially medulla.

1
2
3
4

5

6

Bond and Orr.

7

8

9

were plenty of follicles, but only the nucleated oöcyte was in a primordial follicle, and its staining was poor.

Thereafter, the ovaries were examined histologically in most of the mice. The changes that occurred in the Marsh A and BALB/c ovaries were comparable; the reaction in the C3H mice was somewhat different.

*Marsh A and BALB/c mice.*—After 13 days, stained oöcytes were seen only exceptionally, though follicles persisted for some time (Fig. 5). Mitotic activity was quite considerable in the Graäfian follicles, but it is not possible to say whether they were effective or just suspended. At 19 days and after, there was seen direct luteinisation of the granulosa cells of the follicles, which increased as time went on. At 26 days, a little diffuse luteinisation of the stroma was seen; this had become more pronounced in the mice killed later, i.e. after skin tumours had begun to appear (Fig. 6). There was practically no formation of corpora lutea.

*C3H mice.*—There was loss of oöcytes from the ninth day onwards in the Graäfian follicles, and also to some extent in the primordial follicles, but even at 28 days there were some oöcytes in the latter with stained, albeit karyolytic, oöcytes (Fig. 7). There was also an attempt at the production of stunted corpora lutea. Mitoses and pyknoses were prominent in the membranae granulosae. Diffuse stromal luteinisation was rather greater than in the other two strains.

Though it did not appear from the histology of the oöcytes that they were likely to be viable, it was nevertheless thought wise to test this point biologically by mating the mice surviving after 28 days with males. When we had almost decided that pregnancy was not going to occur, 13 out of 15 mice produced litters at times ranging from 32–64 days from the start of mating. The litters were small, ranging from one to seven, with a mean and standard error of $3{\cdot}2 \pm 0{\cdot}45$. (The average C3H litter in this Institute is seven to eight.) The progeny were small and of poor quality, only 43% surviving to weaning, and many of these dying shortly afterwards. This finding is important, because it shows that the judgement of viability on morphological criteria may be misleading. Unfortunately we have no parallel observations on the other two strains.

Four of the parous mice developed skin tumours—three papillomas and one carcinoma. One had a mammary carcinoma, and one a miliary granulosa-cell tumour of an ovary.

The histology of these ovaries, in general, was different from anything else seen in this experiment. Oöcytes with stained nuclei persisted in small numbers to the end, mostly in primordial follicles. A striking difference from the other strains was the presence in many of these ovaries of large well defined corpora lutea consisting of eosinophilic cells; in some cases the corpora lutea occupied up to two-thirds of the section area. It seems clear, therefore, that the C3H ovary is less vulnerable to DMBA than the ovaries of Marsh A or BALB/c mice.

*Adrenal changes*

It was observed in the first few days of the necropsies on these animals that the adrenals were altered in consistency, showed a loss of the yellow lipoid colour, and were often speckled with petechiae. In view of the observations that have been made by many workers of necrosis after DMBA in *rat* adrenals, it was decided to examine them histologically.

There was a good deal of individual variation, but broadly speaking, disappearance of the medulla, zona reticularis and zona glomerulosa occurred quite quickly, within 7–9 days (Fig. 8). This persisted, and was accompanied by parenchymatous degeneration with loss of fasciculation of the zona fasciculata, but after 19 days there was a progressive recovery, with restoration of the medulla and gradual reconstitution of the glomerulosa and reticularis (Fig. 9). By the time skin tumours appeared, the architecture of the adrenals was more or less normal. The medulla of the C3H adrenal was less vulnerable to DMBA than that of the other strains. At no stage was any manifest necrosis seen.

### DISCUSSION

The findings described confirm in general the previous results (Orr, 1955). The epidermis and hair follicles were destroyed, the former being regenerated by ingrowths from the periphery, and the latter by redifferentiation *de novo* from the new epidermis. In cases where the proximal parts of the hair follicles persisted after DMBA application, probably because they were in the growth phase at the time of treatment, they went on to form inert undifferentiated cysts which underwent absorption with foreign body reaction; they played no part in regeneration or in the formation of tumours.

The part played by hair follicles in carcinogenesis has been much argued about, and Ghadially (1961), for instance, would derive different types of skin tumours severally from the superficial part of the hair follicles, from the deeper part, from superficial epidermis (glabrous or between hair follicles), and from sebaceous glands. Hair follicles and sebaceous glands, at any rate in the mouse, are evanescent structures, and it would seem to us more in accordance with the total facts to relate difference in tumour structure to the retention by the neoplastic epidermis of the capacity to differentiate in various directions.

Other work relevant to these considerations has been discussed in the previous publication (Orr, 1955), but since that time a comprehensive review by Billingham (1958) has considered the evidence for and against the neogenesis of hair follicles. Billingham points out that there is at least one naturally occurring example of hair neogenesis, in the skin covering the antlers of deer. As year by year the antlers are shed and reconstituted, there is a very large area to be covered by new skin containing hair follicles; there is no loss of hair on adjacent parts as would be expected if the antler hairs were the result of migration of existing follicles.

Giovanella and Heidelberger (1967), using an initiating and promoting technique with DMBA and croton oil respectively, found that the incidence of skin tumours was very much lower in hairless mice than in ordinary Swiss mice, although the binding of DMBA to DNA, RNA and protein was identical in the two strains. They concluded that the hair follicles play a major role in skin carcinogenesis. This statement we believe to be broadly true; it is well known that experimental tumours are more easily raised on hairy than on glabrous skin, and the spontaneous skin tumours of, e.g., man rarely appear on completely glabrous skin. But it is the capacity of the skin to produce hair follicles, rather than the formed follicles themselves, which is the operative factor. Evidence is accumulating to indicate that tumours may arise when the ability of the hair follicle to differentiate is impaired because of loss of the dermal papilla rests or some other factor (cf. Wolbach, 1951; Gillman *et al.*, 1955).

*Ovaries and adrenals*

These organs were not examined systematically throughout the experiments, but the sample is sufficiently representative to make it worth while to draw attention briefly to a few points.

The primary effect of DMBA on the ovary of strains BALB/c and Marsh A is destruction of the oöcytes. This corresponds with what was found by Marchant (1957) for three pure strains and outbred albinos. In the present experiments the oöcytes of C3H mice were less vulnerable, and moderately effective breeding was still positive more than a month after DMBA treatment. There was no necrosis of corpora lutea, as has been described in the rat (Wong, Warner and Yang, 1962). Indeed, corpora lutea were quite strikingly absent from our mice except for the post-partum C3H animals. One Marsh A had a macroscopic granulosa-cell tumour, and one C3H a similar tumour detected microscopically, but the experiments were not prolonged enough to know what the incidence of ovarian tumours in these strains might have been.

The mouse adrenal does not show necrosis as has been described for the rat (Huggins and Morii, 1961; Currie, Helfenstein and Young, 1962; Wong *et al.*, 1962). Cefis and Goodall (1965) observed the absence of necrosis in the mouse adrenal (four strains), and stated that with few exceptions the adrenals appeared to be completely normal morphologically. The first of these statements is confirmed in our material, but in all three of our strains there was a loss, without overt necrosis, of all zones of the adrenal except the zona fasciculata. All these zones later recovered, including the medulla, which is of course neural tissue, generally believed to be incapable of regeneration (Dr. W. T. Smith, personal communication). Further comment on this point would be inappropriate in the present context.

### SUMMARY

Mice of three pure lines were painted with one application of approximately 0·75 mg. of 7,12-dimethylbenz(*a*)anthracene (DMBA) in acetone, and histologically examined at intervals up to 28 days thereafter. The surviving mice were painted with croton oil in acetone to establish that the dose of DMBA was effectively initiating.

Macroscopic epilation started at 6 days, and had occurred in all mice by 11 days. Four days after DMBA, the superficial epidermis, distal parts of hair follicles and sebaceous glands were necrotic or destroyed. Regeneration of the epidermis took place by ingrowth from the surrounding untreated skin from 4 days onwards. From this regenerated epidermis new hair follicles were differentiated *de novo*. When the proximal part of the hair follicles survived, it formed cysts which underwent degeneration and absorption with foreign body reaction.

Tumours arose from the superficial epidermis; the hair follicles played no part, and none of the tumours showed trichoepitheliomatous structure.

Mast cells were associated with hair follicles, and were abundant in the stroma of papillomas, but not of carcinomas.

The ovaries showed loss of oöcytes in two of the strains from 6 days onwards; no recovery took place. In the C3H strain oöcytic destruction was not so complete; biological testing showed them still viable 28 days and more after DMBA.

The adrenal showed loss of medulla, zona reticularis and zona glomerulosa at from 7–9 days. Recovery took place from 19 days onwards; at no stage was there overt necrosis.

We would like to thank Dr. W. L. Simpson, who made this collaboration possible, for his interest and helpfulness. Dr. Philippe Shubik kindly let us have a supply of croton oil. Dr. Suzanne R. Salva gave us valuable advice about mast cells from her extensive experience of the subject. The work was supported in part by N.I.H. Grant FR 5529, in part by Grant CA-2903 from the National Cancer Institute, and in part by an Institutional Grant from the United Foundation of Greater Detroit.

### REFERENCES

BILLINGHAM, R. E.—(1958) In 'The Biology of Hair Growth'. Edited by William Montagna and Richard A. Ellis. New York (Academic Press, Inc.), p. 451.
CEFIS, F. AND GOODALL, C. M.—(1965) *Am. J. Path.*, **46**, 227.
CURRIE, A. R., HELFENSTEIN, J. E. AND YOUNG, S.—(1962) *Lancet*, ii, 1199.
GHADIALLY, F. N.—(1961) *Cancer, N.Y.*, **14**, 801.
GILLMAN, T., PENN, J., BRONKS, D. AND ROUX, M.—(1955) *Br. J. Cancer*, **9**, 272.
GIOVANELLA, B. C. AND HEIDELBERGER, C.—(1967) *Proc. Am. Ass. Cancer Res.*, **8**, 21.
HUGGINS, C. AND MORII, S.—(1961) *J. exp. Med.*, **114**, 741.
MARCHANT, JUNE—(1957) *Br. J. Cancer*, **11**, 452.
ORR, J. W.—(1955) *Br. J. Cancer*, **9**, 623.
WONG, TING-WA, WARNER, NANCY AND YANG, N. C.—(1962) *Cancer Res.*, **22**, 1053.
WOLBACH, S. B.—(1951) *Ann. N.Y. Acad. Sci.*, **53**, 517.